# Left Ventricular Noncompaction Cardiomyopathy in Children: A Focus on Genetic and Molecular Mechanisms

**DOI:** 10.31083/RCM39044

**Published:** 2025-08-29

**Authors:** Monica B. Lehman, Buyan-Ochir Orgil, Karine Guerrier, Keiichi Hirono, Enkhzul Batsaikhan, Kazuyoshi Saito, John W. Collyer, Jeffrey A. Towbin, Enkhsaikhan Purevjav

**Affiliations:** ^1^The Heart Institute, Department of Pediatrics, University of Tennessee Health and Science Center, Memphis, TN 38103, USA; ^2^Children's Foundation Research Institute, Le Bonheur Children's Hospital, Memphis, TN 38103, USA; ^3^Department of Pediatrics, Graduate School of Medicine, University of Toyama, 930-8555 Toyama, Japan; ^4^Department of Pharmacology, Toxicology and Addiction Sciences, University of Tennessee Health and Science Center, Memphis, TN 38103, USA; ^5^Department of Pediatrics, School of Medicine, Fujita Health University, 470-1192 Aichi, Japan; ^6^Department of Pediatrics, School of Medicine, University of Pittsburgh, Pittsburgh, PA 15260, USA; ^7^Cardiology, St. Jude Children’s Research Hospital, Memphis, TN 38105, USA

**Keywords:** noncompaction, cardiomyopathy, heart failure, gene mutation, fetal heart development

## Abstract

Left ventricular noncompaction (LVNC), also called noncompaction cardiomyopathy (NCM), is a myocardial disease that affects children and adults. Morphological features of LVNC include a noncompacted spongiform myocardium due to the presence of excessive trabeculations and deep recesses between prominent trabeculae. Incidence and prevalence rates of this disease remain contentious due to varying clinical phenotypes, ranging from an asymptomatic phenotype to fulminant heart failure, cardiac dysrhythmias, and sudden death. There is a strong genetic component associated with LVNC, and nearly half of pediatric LVNC patients harbor an identifiable genetic mutation. Recent studies have identified LVNC-associated mutations in genes involved in intercellular trafficking and cytoskeletal integrity, in addition to well-known mutations causing abnormal cardiac embryogenesis. Currently, the diagnosis is based on symptoms, as well as various diagnostic criteria, including echocardiography, electrocardiograms, and cardiac magnetic resonance imaging. Meanwhile, clinical management is primarily focused on the prevention of complications, such as heart failure, thromboembolic events, life-threatening arrhythmias, and stroke. Continued research is focusing on the genetic etiology, the development of gold-standard diagnostic criteria, and evidence-based treatment guidelines across all age groups. This review article will highlight the genotype–phenotype relationship within pediatric LVNC patients and assess the latest discoveries in genetic and molecular research aimed at improving their diagnostic and therapeutic management.

## 1. Introduction

A cardiomyopathy is a disease of the myocardium that causes systolic dysfunction, diastolic dysfunction, or an increased propensity for arrhythmias. Left ventricular noncompaction (LVNC), also called noncompaction cardiomyopathy (NCM), results from abnormal myocardial maturation and compaction and is a classified form of cardiomyopathy in the United States [1]. Per the American Heart Association’s 2019 statement on cardiomyopathy in children, LVNC meets the classification as a congenital cardiomyopathy in pediatric patients and presents in isolation, including those with normal systolic function (isolated form), or alongside characteristics seen in other cardiomyopathies (non-isolated form) [2]. Non-isolated forms of LVNC can be subdivided into a dilated cardiomyopathy (DCM) phenotype, a hypertrophic cardiomyopathy (HCM) phenotype, an arrhythmogenic cardiomyopathy (ACM) phenotype, or a restrictive cardiomyopathy (RCM) phenotype. In addition, some patients may have a right ventricle (RV) only phenotype, a biventricular phenotype, an undulating cardiomyopathy phenotype (meaning the phenotype starts as one phenotype—such as DCM with hyper-trabeculation—and then changes to an HCM with hyper-trabeculation and back to the DCM with hyper-trabeculation phenotype) [3, 4]. The congenital heart disease (CHD) phenotype is a co-morbidity in patients with LVNC and CHD [5, 6]. In contrast, the European Society of Cardiology identifies LVNC as an “unclassified” cardiomyopathy or morphological trait shared by phenotypically distinct cardiomyopathies [7]. Although there is a divergence in characterizing LVNC as a normal variation of fetal heart development, a distinct genetic cardiomyopathy, or an acquired morphological trait associated with other types of cardiomyopathies, structural features of this entity are broadly recognized among experts [3, 8, 9].

**Fig. 1.**
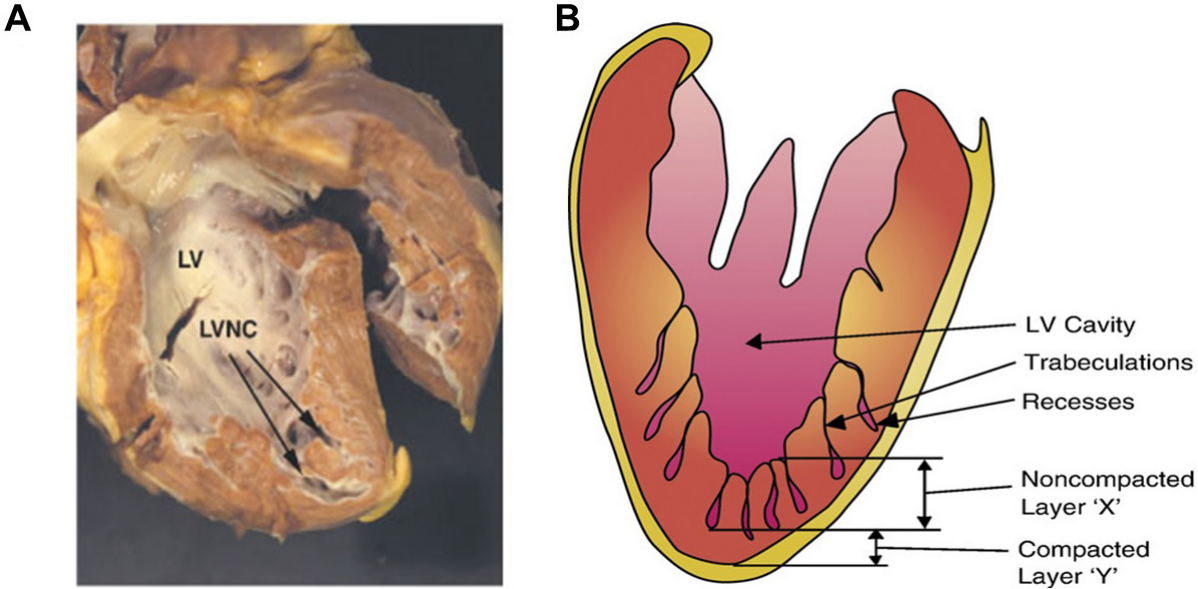
**Morphopathological appearance of left ventricular noncompaction (LVNC)**. (A) An autopsy image of the heart seen as a spongy myocardium. Arrows indicate trabeculations towards the apex and left ventricle (LV) lateral walls. (B) Schema of noncompacted and compacted layers of the LV walls with deep trabeculations and intertrabecular recesses. Adapted with permission from Towbin and Bowles [11]. The failing heart. *Nature* 415, 227–233 (2002). https://doi.org/10.1038/415227a.

Morphologically, LVNC has two distinct layers within the LV myocardium: the spongy, noncompacted meshwork and the thin compacted layer mainly seen at the apical region of the heart [10, 11]. The “spongy” myocardial meshwork includes extensive trabeculae and deep recesses between trabeculae carneae that provide a potential source for severe cardiac complications, such as thrombosis, arrhythmias, cardiac arrest, and heart failure (Fig. 1, Ref. [11]). Additionally, the RV may be affected in isolation or in combination with the LV, leading to isolated LV, RV, or biventricular heart failure [5, 12, 13, 14].

Genetically, LVNC is heterogeneous, and 45% of the affected pediatric population have identified genetic mutations [14, 15]. Among adults, LVNC is a rare diagnosis and 30% of the affected adult population have an identified genetic mutation or chromosomal abnormality [9, 12, 13, 16, 17]. Despite a strong association with genetic abnormalities, a direct genotype-phenotype relationship has yet to be established in many LVNC cases [4]. This is partially due to diverse clinical and pathological phenotypes of LVNC in patients of all ages, including acquired noncompaction cases of various etiologies and speculative modifier factors. Another challenge in identifying the genotype-phenotype relationship is the diversity of identified genetic mutations in causal and modifier genes [14, 18, 19]. Recent studies have utilized whole exome sequencing among affected family members to further investigate the complex genotype-phenotype relationships of ventricular noncompaction [19, 20, 21, 22].

This review article will broadly outline recent improvements in the diagnosis and management of this disease in pediatric patients with an emphasis on underlying genetic and molecular factors in the development of LVNC.

## 2. Epidemiology 

The first cases of isolated LVNC without cardiac malformations were described in the 1990s [23, 24] and because of advances in echocardiography and cardiac magnetic resonance (CMR) imaging, the ability to diagnose LVNC has improved, enabling better diagnostic accuracy and leading to an increasing rate of patients currently identified with LVNC. Despite thirty years of progress, the true estimation of the incidence and prevalence of LVNC remains challenging due to the heterogeneous nature of the disease, varying diagnostic criteria, and a tendency for hypertrabeculation and noncompaction of the myocardium in high-risk populations, such as patients with CHD, heart failure or other cardiac and non-cardiac morbidities, and stresses [25, 26, 27, 28, 29]. Adults without heart failure have shown to develop hypertrabeculation as an adaptive response to physiological stress. This phenomenon has been demonstrated in adult athletes, pregnant women, and patients with sickle cell anemia, skeletal myopathies, and chronic renal failure [8, 30]. Unlike hypertrabeculation due to physiological stress, LVNC caused by genetic mutation will not fully resolve once the physiological stress is removed [14].

Approximately 5% of pediatric cardiomyopathy patients have been diagnosed with LVNC compared with 3% to 4% of adult patients who have heart failure with associated LVNC [18, 31, 32]. A recent pediatric study showed nearly 9% of all cardiomyopathy cases are now diagnosed with LVNC, recognizing it as the third most common form of inherited cardiomyopathies in children [2]. Per the Pediatric Cardiomyopathy Registry (PCMR), LVNC has a familial inheritance pattern of up to 40% with estimated occurrence of ~1 per 7000 live births [1, 16, 32]. The reported ratio of isolated to non-isolated forms of LVNC is 6:1 [2]. Recently, a prevalence of LVNC has been estimated as of 0.076% in a population-based cohort of unremarkable neonates by echocardiography [12], while the estimated prevalence in middle and high school students was 17.5% based on CMR screening [33]. Both studies demonstrated that LVNC was associated with lower parameters in systolic function and with an increased risk of LV dysfunction, even if clinically asymptomatic.

## 3. Genetic Etiologies and Genotype-Phenotype Associations

Genetic etiologies of isolated primary LVNC are heterogeneous, although the genetic basis is still unresolved in most LVNC patients. While gene defects are identified in only 30% of adult patients with LVNC [15], a familial trait is evident in approximately 40% of infants with LVNC being the dominant cases with incomplete penetrance of autosomal dominant, autosomal recessive, or X-linked inheritance patterns [3]. In some cases, mitochondrial inheritance is noted. A genome-wide linkage analysis in families with autosomal dominant LVNC identified the associated genetic *loci* on chromosome (Chr) 11p15 and 8p23.1. The Chr.11 locus includes cardiomyopathy-associated genes, such as glycine-rich protein (*CSRP3*/*MLP*) and SRY-Box Transcription Factor 6 (*SOX6*) [34], while an interstitial deletion of the Chr.8p23.1 contains GATA binding protein 4 (*GATA4*), a zinc-finger transcription factor involved in the cardiac embryogenesis [35]. Other specific gene mutations occur in a relatively small number of genes (Table 1, Ref. [18, 20, 34, 35, 36, 37, 38, 39, 40, 41, 42, 43, 44, 45, 46, 47, 48, 49, 50, 51, 52, 53, 54, 55, 56, 57, 58, 59, 60, 61, 62, 63, 64, 65, 66, 67, 68, 69, 70, 71, 72, 73, 74, 75, 76])—including cardiac α-actin (*ACTC1*), bone morphogenetic protein 10 (*BMP10*), myosin binding protein C (*MYBPC3*), β-myosin heavy chain (*MYH7*), MIB E3 ubiquitin protein ligase 1 (*MIB1*), alpha-dystrobrevin (*DTNA*), α-tropomyosin (*TPM1*), lim domain binding 3 (*LDB3*), PR domain containing 16 (*PRDM16*), and cardiac troponin T (*TNNT2*)*—*have been linked with noncompaction phenotypes in humans and mouse models to date [10, 14, 36, 77]. LVNC patients with heart failure have demonstrated a high rate of pathologic variants in *TTN* (titin) and *SCN5A* (sodium channel protein type 5 subunit alpha), supporting the notion that these genes are implicated in the development of LVNC as disease-causing or disease-modifying genes [37, 38]. Evidence shows an increased burden of variants in ion channel genes, such as *SCN5A*, *ANK2*, *CACNA1C*, *ABCC9*, *HCN4*, *KCNH2*, *KCNE3*, *KCNQ1*, *RYR1*, and *RYR2* in pediatric LVNC patients as reported by Hirono *et al*. [78]. Multiple studies have demonstrated oligogenic or multigenic inheritance among families with LVNC [9, 19, 79]. A key study from the Netherlands demonstrated that 41% of adult and pediatric probands had an identified genetic mutation, and familial screening revealed their affected relatives were largely asymptomatic at the time of diagnosis [80]. Discovering LVNC in asymptomatic individuals illustrates the importance of genetic testing of probands and their relatives for all families with isolated and non-isolated LVNC subtypes.

**Table 1.** **Genes associated with left ventricular noncompaction (LVNC) or noncompaction cardiomyopathy (NCM)**.

Chromosome	LVNC or NCM Genes [References]	Additional Cardiomyopathy Phenotypes [References]
Chr11p15	*MLP; SOX6* [34]	DCM [39], HCM [40, 41]
Chr8p23	*GATA4* [35]	CHD [42]
Chr15q14	*ACTC1* [43, 44]	DCM [45], HCM [46], CHD [47]
Chr1q43	*ACTN2* [20]	DCM [39], HCM [48]
Chr18q11	*MIB1* [49]	
Chr11q11	*MYBPC3* [50]	DCM [51], HCM [52]
Chr14q11	*MYH7* [53]	HCM [54], CHD [55]
Chr15q22	*TPM1* [56]	DCM [57], HCM [58], CHD [56, 59]
Chr18q12	*DTNA* [18]	
Chr2q35	*DES* [60]	DCM [61]
Chr2p13	*BMP10* [36]	
ChrXq24	*LAMP2* [62, 63]	DCM [64], HCM [63]
Chr10q23	*LDB3* [65, 66]	DCM [67], HCM [48]
ChrXq28	*TAZ* [18]	DCM [68]
Chr2q31	*TTN* [38]	ACM [69], DCM [70], HCM [71]
Chr3p22	*SCN5A* [37]	DCM [72], HCM [73]
Chr10q22	*VCL* [74]	HCM [74, 75], DCM [76]

ACM, arrhythmogenic cardiomyopathy; CHD, congenital heart disease; DCM, dilated cardiomyopathy; HCM, hypertrophic cardiomyopathy; NCM, noncompaction cardiomyopathy; *MLP*, glycine-rich protein; *SOX6*, SRY-Box Transcription Factor 6; *GATA4*, GATA binding protein 4; *ACTC1*, cardiac α-actin; *MIB1*, MIB E3 ubiquitin protein ligase 1; *MYBPC3*, myosin binding protein C; *MYH7*, β-myosin heavy chain; *TPM1*, α-tropomyosin; *DTNA*, alpha-dystrobrevin; *DES*, desmin; *BMP10*, bone morphogenetic protein 10; *LAMP2*, Lysosome-associated membrane glycoprotein 2; *LDB3*, lim domain binding 3; *TAZ*, tafazzin; *TTN*, titin; *SCN5A*, sodium channel protein type 5 subunit alpha; *VCL*, vinculin.

**Fig. 2.**
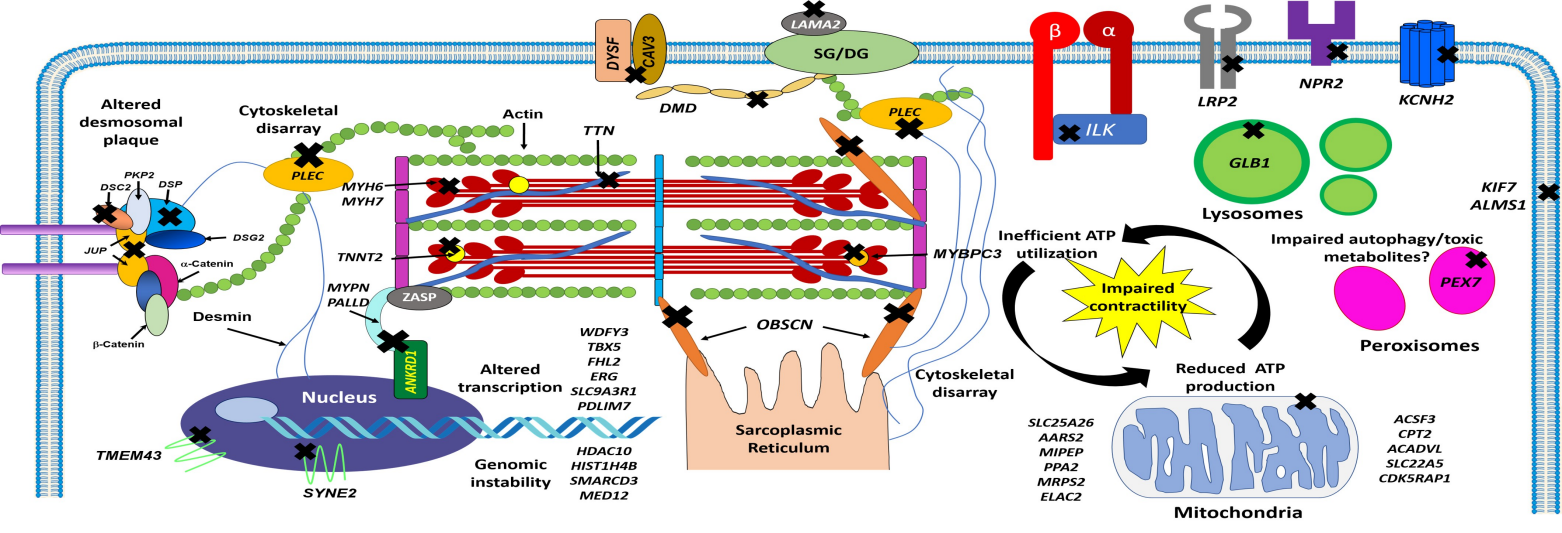
**Schematic representation of cardiomyocyte structure and location of genes implicated with LVNC phenotypes and molecular pathways involved**. Cross symbols indicate mutated genes that encode cytoskeletal, ion channel, nuclear, sarcomere, and sarcoplasmic proteins associated with LVNC. ATP, adenosine triphosphate; *DSC2*, desmocollin 2; *DSP*, desmoplakin; *DSG2*, desmoglein 2; *JUP*, junctional plakoglobin; *PKP2*, plakophilin 2; *TMEM43*, transmembrane protein 43; *SYNE2*, nesprin 2; *ANKRD1*, ankyrin repeat domain 1; *MYPN*, myopalladin; *PALLD*, palladin; *TNNT2*, cardiac troponin T; *DMD*, dystrophin; *DYSF*, dysferlin; *CAV3*, caveolin 3; *LAMA2*, laminin subunit alpha 2; *SG/DG*, sarcoglycans/dystroglycans; *PLEC*, plectin; *ILK*, integrin-linked kinase; *LRP2*, LDL receptor related protein 2; *NRP2*, neuropilin 2; *KCNH2*, voltage-activated potassium channel; *GLB1*, galactosidase, beta 1; *KIF7*, kinesin 7; *ALMS1*, Alstrom syndrome protein 1; *PEX7*, peroxisomal biogenesis factor 7; *OBSCN*, obscurin; *WDFY3*, WD repeat and FYVE domain-containing protein 3; *TBX5*, T-box protein 5; *FHL2*, four-and-a-half LIM domain protein 2; *ERG*, ETS-related gene; *SLC9A3R1*, sodium-hydrogen antiporter 3 regulator 1; *PDLIM7*, PDZ and LIM domain protein 7; *HDAC10*, histone deacetylase 10; *HIST1H4B*, histone H4; *SMARCD3*, SWI/SNF-related matrix-associated actin-dependent regulator of chromatin subfamily D member 3; *MED12*, mediator complex subunit 12; *SLC25A26*, solute carrier family 25 member 26; *AARS*, alanyl-tRNA synthetase; *MIPEP*, mitochondrial intermediate peptidase; *PPA2*, protein phosphatase 2A; *MRPS2*, mitochondrial ribosomal protein S2; *ELAC2*, ElaC ribonuclease Z2; *ACSF3*, acyl-CoA synthetase family member 3; *CPT2*, carnitine palmitoyltransferase 2; *ACADVL*, acyl-CoA dehydrogenase very long chain; *SLC22A5*, solute carrier family 22 member 5; *CDK5RAP1*, CDK5 regulatory subunit-associated protein 1.

As shown schematically in Fig. 2, the LVNC-associated genes identified to date are largely involved in sarcomere function, mitochondrial function, regulation of transcription and translation, protein degradation, ion channel function, and signal transduction. More recently, genes that encode proteins involved in intercellular trafficking, cellular junction, and cytoskeletal integrity of the myocardium have also been identified [15, 36, 78, 81].

Non-isolated LVNC phenotypes with extended clinical variability in young children have been associated with mitochondrial disorders, such as Barth syndrome, which is caused by *TAZ/G4.5* (tafazzin) mutations, zaspopathy-caused mutations in *ZASP*, hereditary neuromuscular disorders, chromosomal defects (such as 1p36, 1q43, and distal 5q deletions), Turner syndrome, Ohtahara syndrome, trisomy 22, trisomy 13, and DiGeorge syndrome [6, 9, 18, 65, 82, 83, 84]. The genotype-phenotype correlation is well identified between LVNC and X-linked Barth syndrome [18, 24]. LVNC has also been associated with a variety of CHDs, such as multiple, small ventricular septal defects, bicuspid aortic valve, and Ebstein’s anomalies [28, 29]. These coexisting CHDs may also explain the existence of common pathogenic pathways in the maldevelopment of the ventricular myocardium [16, 85, 86].

Acquired LVNC in adult patients has various and speculative etiologies [30]. Incidences of acquired LVNC has been demonstrated in athletes, patients with sickle cell anemia, skeletal myopathies and chronic renal failure, and in pregnant women [8]. It is speculated that acquired ventricular hypertrabeculation in athletes, predominantly in the LV apex, allows for increased compliance, which reduces wall stress and strain [87]. Hypertrabeculation in adult patients with progressive neuromuscular disorders occurs as a part of myocardial remodeling or it may be acquired to increased cardiac pre-load and pressure overload or myocardial damage [88, 89]. It may also be associated with disturbances in desmosomes and activation of WNT signaling that results in the development of ACM [83, 90]. The difference between physiological hypertrabeculation responses and the pathological disease of LVNC is the presence of ventricular dysfunction or fibrosis, cardiac symptoms, and a family history of cardiomyopathy.

## 4. Pathogenesis and Molecular Signaling 

Formation of the normal ventricular wall is based on anatomically overlapping morphogenetic events: trabeculation and compaction of the developing cardiac muscles [91]. During fetal development, ventricular myocardium is initially composed of trabeculations and deep intertrabecular recesses. At approximately week 5 and 8 in human embryonic development, cardiac muscle undergoes gradual compaction, which starts from the epicardial towards the endocardial surface at the base of the heart. As it progresses inward and distally, the LV apex is the last area to undergo compaction [92]. It has been speculated that an abnormal termination of the myocardial compaction process during early development leads to excessive trabeculations with intertrabecular recesses between trabeculae and a spongy noncompacted LV myocardial appearance, as shown in Fig. 1 [93, 94]. This process can also occur in the RV. As noted earlier, the study has also identified genetic mutations associated with intercellular trafficking and cytoskeletal integrity, among others (Fig. 2), which may indicate more complex polygenetic interactions lead to the development of LVNC’s distinct morphological features [15].

Animal studies have demonstrated that normal trabeculation and compaction processes depend on an exquisite balance between cardiomyocyte proliferation, differentiation, and maturation [91, 93, 95, 96]. In mice, the trabeculation process starts at E8.0–8.5 when endothelial cells sprout towards the myocardium, forming endocardial domes filled with cardiac jelly or the so-called “extracellular matrix (ECM) bubble” with primitive cardiomyocytes proliferating into laminar trabeculae [97]. Further, trabeculae undergo assembly, extension and growth followed by the termination process occurring at around E14.5. Concomitant with trabecular growth, ECM bubbles are progressively reduced from the basal parts to the apex of embryonic heart. Myocardial compaction process occurs at the base of trabeculae adjacent to the outer myocardium, forming the compacted ventricular muscle wall [98]. The development of trabeculae is vigorously controlled by a disintegrin and metalloproteinase with a thrombospondin motif 1 (*ADAMTS1*) protease that digests ECM proteoglycan versican in the heart [99]. In normal cardiac embryogenesis, *ADAMTS1* expression in the cardiac jelly is suppressed by brahma-related gene 1 (*BRG1*)-mediated chromatin remodeling, and suppression of *ADAMTS1* protease is critical for completion of trabecular growth [100]. Later in the maturating heart, *ADAMTS1* expression is de-repressed (initiated) in the endocardium; its activation degrades the cardiac jelly, preventing excessive hyper-trabeculation within the adjacent myocardium, as seen in LVNC resulting from the failure of termination of ADAMTS1-mediated trabeculation caused by a single mutation in the *CHD4* gene that encodes chromodomain helicase DNA-binding protein 4 [101].

Signaling pathways such as NOTCH, NRG1, BMP, and Nkx2-5 have been shown to play critical roles for balanced processes of normal trabeculation and compaction [49, 97, 102, 103, 104, 105, 106]. NOTCH is a highly evolutionary conserved signaling pathway involving transmembrane receptors (NOTCH 1–4) with extracellular and intracellular domains that interact with the ligands (Delta-like1, 3, 4, and Jagged1, 2) that control cell fate, differentiation, and patterning [107, 108, 109]. In the cardiovascular system, development of ventricular myocardium and coronary vessels is mediated by NOTCH1, and communication between the endocardium and myocardium during cardiomyocyte proliferation and differentiation is tightly regulated by NOTCH signaling [110]. It has been shown that NOTCH activity within the developing endocardium is regulated by JARID2 [93, 111], a transcriptional repressor of several cardiac transcriptional factors, including Nkx2.5, GATA4 [112], MEF2 [113], retinoblastoma protein (RP), and cyclin D1 [114, 115].

**Fig. 3.**
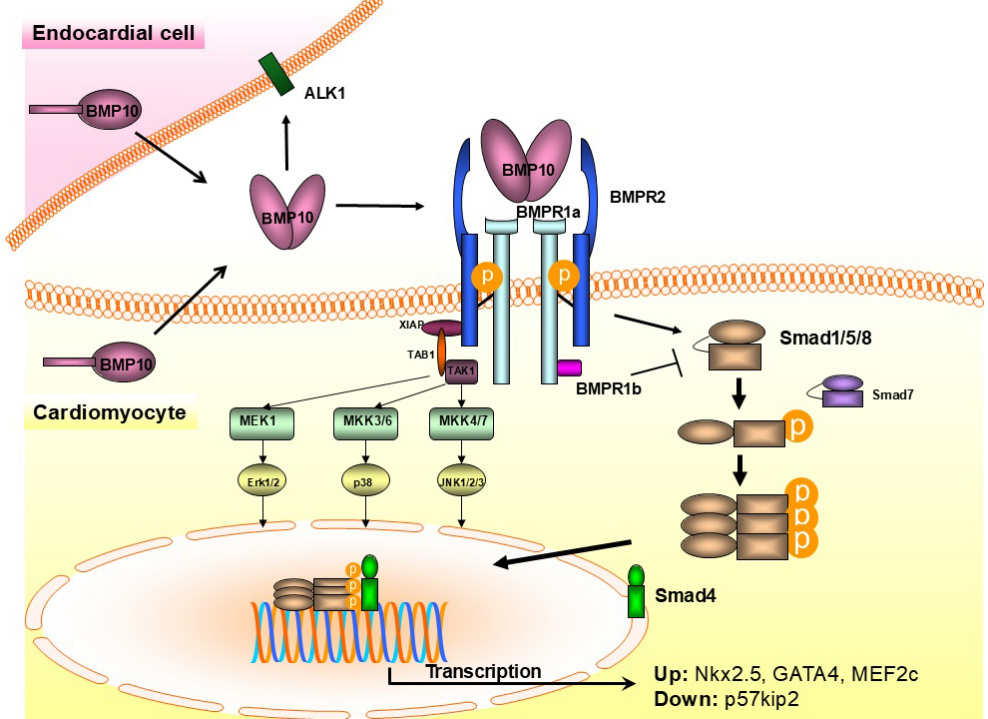
**Schema of BMP10-mediated signaling in endocardial and myocardial cells**. BMP10, bone morphogenetic protein 10; BMPR, BMP receptor; ALK1, activin receptor-like kinase 1; XIAP, X-linked inhibitor of apoptosis; TAK1, transforming growth factor beta (TGF-β)-activated kinase 1; TAB1, TAK1-binding protein 1; MEK1, mitogen-activated protein kinase kinase; MKK, mitogen-activated protein kinase; ERK, extracellular signal-regulated kinase; p38, protein 38; JNK, c-Jun N-terminal kinase; Smad, suppressor of mother against decapentaplegic; Nkx2.5, NK2 homeobox 5; myocyte-specific enhancer factor 2C; p57kip2, cyclin-dependent kinase inhibitor 1C.

**Fig. 4.**
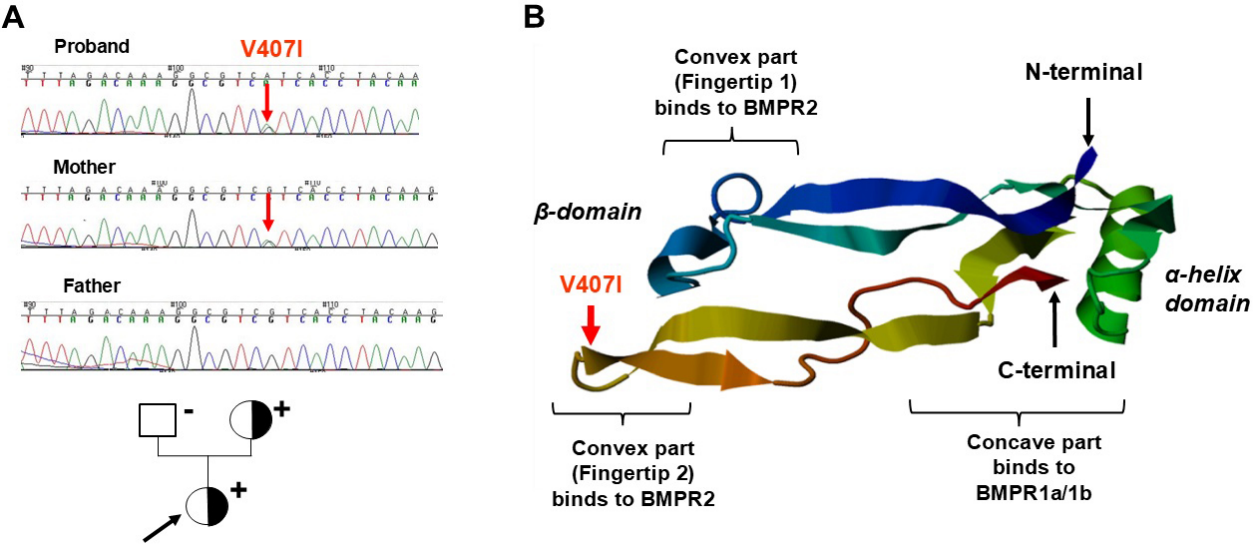
**Chromatographic images of sequencing in the members of the LVNC family (left) and the structure of the BMP10 protein (right)**. (A) Chromatographs of direct sequencing (upper panels) and the pedigree of the family with LVNC (lover panel). The proband indicated by black arrow and affected mother with LVNC carried the c.1219G>A (V407I) variant in the BMP10 gene. (B) Structure of the BMP10 protein. The mature BMP10 forms two domains, alpha and beta. The beta-domain has two finger-shaped convex parts, which binds to BMPR2. Red arrows indicate the V407I mutation and its location in the β-domain Fingertip2 of BMP10. Adapted with permission from Hirono *et al*. [36] Familial left ventricular non-compaction is associated with a rare p.V407I variant in bone morphogenetic protein 10. Circ J. 2019 Jul 25;83(8):1737–1746. https://doi.org/10.1253/circj.CJ-19-0116.

NOTCH also controls the expression of BMP10, a peptide growth factor in the TGF-β family that functions as the key regulator of ventricular trabeculation and compaction [108, 116, 117, 118]. In mouse embryos, expression of BMP10 is documented in the ventricular myocardium from E9.0 to E13.5 and in the atria from E16.5 to E18.5, suggesting a crucial role for BMP10 in myocardial maturation [36]. Three specific BMP receptors (BMPRs) have been identified on endocardial and myocardial cells (Fig. 3), including BMPR1a or activin receptor-like kinase 3 (ALK3), BMPR1b (ALK6), and BMPR2 [108]. The C-terminus of BMP10 binds to BMPR1a and BMPR1b, while two fingertip domains (Fingertip1 and Fingertip2) in the β-domain of BMP10 bind to the BMPR2 (Fig. 4, Ref. [36]).

As shown in Fig. 3, BMP10’ binding to its BMPRs regulates cardiomyocyte proliferation and trabeculation during cardiogenesis *via* activation of SMAD1/5/8 (canonical) and MAPK (non-canonical) as a result of phosphorylation (p), which downstream activate pathways involve NKX2-5, MEF2c, and TBX20 cardiogenic factors [93, 117], but inhibiting CDKN1c/p57-kip2 [119]. A pathogenic *BMP10* mutation (c.1219G>A, p.V407I) has recently been identified in familial LVNC cases, a proband, and her affected mother [36]. The V407I mutation located in the Fingertip2 domain of BMP10 (Fig. 4) altered the interaction of BMP10 with receptors, BMPR1a and BMPR2. Subsequently, abnormal cytoplasmic aggregations of BMP10 in cardiomyocytes, inhibition of the proliferation of differentiating H9C2 cardiac myoblasts, and cellular intolerance to cyclic stretch have been demonstrated [36].

*Mib1*, another NOTCH pathway element, is associated with the biventricular noncompaction phenotype, ventricular dilatation, and heart failure when mutated [10]. Genetic testing of 100 European patients identified V943F and R530X variations in *MIB1*. Injection of *Mib1*-mutant V943F and R530X mRNAs into zebrafish embryos disrupted Notch signaling and reduced myocardial arrest producing immature trabeculae and noncompaction [49]. In LVNC cases with the associated CHD, interruption of the NOTCH or WNT signaling appears to be part of a “common final pathway” of this form of LVNC [9, 97, 108]. LVNC and ACM also have overlapping associations with WNT signaling disturbances [83, 90].

## 5. Diagnostic Testing 

Adult and pediatric LVNC patients are commonly diagnosed by imaging at the time of clinical presentation. Echocardiography is commonly used to diagnose a noncompacted ventricular myocardium in patients with LVNC [16, 120]. Echocardiographic criteria for diagnosing LVNC consists of a noncompacted to compacted myocardium ratio of greater than 2:1 in at least one ventricular segment in end-diastole. The apical, mid-septal, and mid-lateral ventricular segments are typically involved [121]. Other studies define LVNC based on the noncompacted to compacted myocardium ratio being greater than 2:1 in end-systole [27, 33]. Cases of increased trabeculations echocardiographic criteria for LVNC during pregnancy with complete or marked resolution of LV trabeculations postpartum have also been documented [122].

CMR imaging with late gadolinium enhancement (LGE) testing is suggested for adult and pediatric patients with suspected LVNC on electrocardiogram (ECG) for precise clinical assessment of noncompaction, myocardial fibrosis, and damage to predict severity of the disease [123, 124]. Diagnostic criteria of LVNC using CMR imaging also varies among studies, although this method is particularly useful for adults in providing more reliable assessment of hypertrabeculation in the apex [125]. Examples of CMR diagnostic criteria include the trabeculated mass being greater than 20% of the global LV mass in end diastole, and an end-diastolic ratio of noncompacted to compacted myocardium greater than 2.3:1 in the short and long axis views [126]. Cases of increased trabeculations with *de novo* echocardiographic criteria for LVNC during pregnancy with complete or marked resolution of LV trabeculations postpartum have also been documented [122]. Moreover, LV strain parameters on CMR imaging were lower in adolescent children and young adults with LVNC compared to healthy age-matched control individuals [127].

**Fig. 5.**
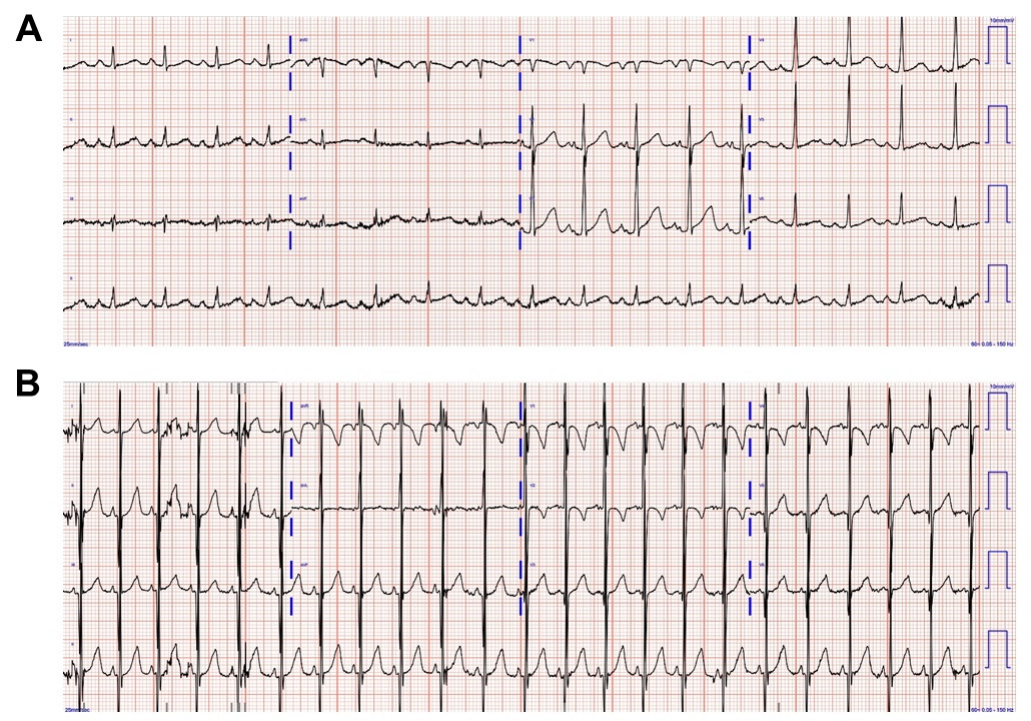
**Electrocardiogram images of pediatric patients with left ventricular noncompaction**. (A) A 12-lead electrocardiogram in a 1-year-old patient with LVNC with sinus rhythm, left atrial enlargement, prolonged PR interval, Q wave in V1, and prolonged QTc interval. (B) A 12-lead electrocardiogram in an infant with LVNC with sinus rhythm, excessive QRS voltage, and biventricular hypertrophy.

ECG is abnormal in 75–94% of pediatric and adult LVNC patients [128]. Typical ECG findings include prolonged QTc intervals, R wave notching, T wave inversion, pathologic Q waves, left axis deviation, and severe LV hypertrophy with gigantic QRS complexes, especially in neonates [94]. In babies with LVNC, the ECG commonly shows extreme QRS complex voltage (Fig. 5). In adults, in addition to all those ECG features, intraventricular conduction delay with predominant left bundle branch block and life-threatening ventricular arrhythmias, such as ventricular tachycardia, and ventricular fibrillation have been reported by Steffel *et al*. [129]. Presence of fragmented QRS complex (fQRS) on ECGs in adult LVNC patients were identified as a novel predictor of arrhythmic events, sudden cardiac death, and mortality [130]. Atrial fibrillations are also associated with LVNC, which may be due to proarrhythmic substrate from the continuity between extensive intertrabecular recesses and endocardium as demonstrated in previous studies [131].

## 6. Clinical Manifestations 

Adults and children with LVNC present with the full spectrum of this heterogenous disease with clinical manifestations ranging from asymptomatic hypertrabeculation to infantile cardiac muscle disease presentation of heart failure with reduced EF (HFrEF) and unfavorable long-term prognoses. As previously reported, adult patients may be asymptomatic and develop hypertrabeculation as a physiological response. These cases are not typically associated with systolic or diastolic dysfunction [8, 30]. It has also been demonstrated that pediatric patients with isolated LVNC and normal ventricular function remain asymptomatic throughout adulthood and into old age [132]. This phenotype accounts for nearly 35% of LVNC cases and has been termed a “benign form” by Towbin *et al*. [3, 5].

Affected adult and pediatric patients typically present with symptoms of chest pain, dyspnea, palpitations, syncope, peripheral edema, or exercise intolerance [9, 78]. Their overall presentation may consist of congestive heart failure, arrhythmias, thromboembolism, embolic ischemic stroke, myocardial infarction, or sudden death [19, 23, 32, 120, 133]. Although some adult patients have adaptive hypertrabeculation, they may also present with heart failure secondary to LVNC. One case study highlighted a 41-year-old heart failure patient with LVNC confirmed by ECG and CMR. Ventricular remodeling was demonstrated after initiation of heart failure guideline-mediated medical therapy [134]. Other case studies diagnosed via ECG and CMR include a previously healthy 62-year-old patient who presented with palpitations and diagnosed with atrial fibrillation [135] and a 78-year-old patient with history of ischemic cardiomyopathy and end-stage renal disease [136]. Both patients started heart failure medications and anticoagulation prophylaxis. Lastly, a 55-year-old patient who presented with dyspnea, chest pain, and peripheral edema was diagnosed with LVNC and right-sided aortic arch. This patient later died from the known complication of ventricular fibrillation [131].

**Fig. 6.**
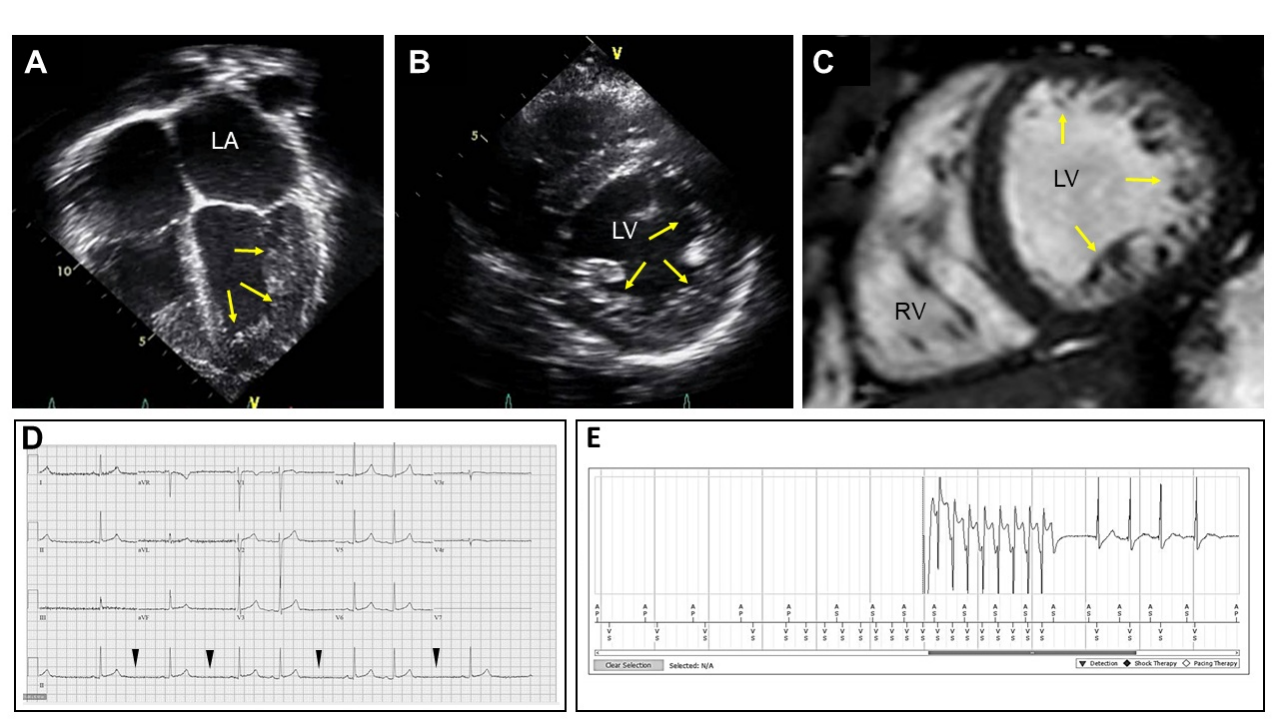
**Results of echocardiographic, cardiac magnetic resonance (CMR) imaging, and electrocardiography (ECG) tracing analyses in a 17-year-old adolescent patient with LVNC**. A representative echocardiography image in four chamber view. Yellow arrows indicate noncompacted myocardial walls with trabeculae and recesses seen in the LV. (A) A parasternal short axis view of echocardiogram demonstrating circumferential noncompaction on the LV walls. (B) A representative CMR image demonstrating circumferential LV noncompaction. (C) An ECG tracing recorded prior to pacemaker placement. Sinoatrial (SA) nodal exit block (Mobitz II) is demonstrated. Arrowheads demarcate timing of SA node exit block. (D) An ECG tracing recorded after pacemaker implantation. (E) Non-sustained ventricular tachycardia found on device interrogation is demonstrated. Adapted with permission from Collyer *et al*. [19] Combining whole exome sequencing with *in silico* analysis and clinical data to identify candidate variants in pediatric left ventricular noncompaction. Int J Cardiol. 2022 Jan 15:347:29–37. https://doi.org/10.1016/j.ijcard.2021.11.001.

LVNC may also have associated CHDs, neuromuscular disorders, or chromosomal defects [6, 9, 15, 23, 81, 137]. Children are more likely to have associated CHD and an identified genetic mutation than their adult counterparts [138]. Complex clinical phenotypes with concurrent dilated, hypertrophic, restrictive, or arrhythmogenic forms, or those with overlapping phenotypes one or more forms of cardiomyopathy or CHD, are also reported [9]. For example (Fig. 6, Ref. [19]), a 17-year-old patient with circumferential apical hypertrabeculation, no systolic dysfunction, and no LGE on echocardiogram and CMR imaging, respectively, required placement of a pacemaker for sinoatrial (SA) nodal exit Mobitz II block, which was followed by upgrading to a defibrillator system due to development of non-sustained ventricular tachycardia (NSVT) after pacemaker implantation and interrogation [19]. Distinct LVNC phenotypes identified impact diagnostic testing, potential treatments, and overall prognosis in the pediatric LVNC population [2, 5, 9].

LVNC in newborns and infants is most commonly non-isolated (mixed) with worst case outcomes reported, particularly in those with associated systemic and metabolic disorders with or without CHD [5, 9]. Those with overlapping forms of cardiomyopathy [14] have an associated increased risk for heart failure [139]. Therefore, it is imperative to follow these pediatric patients long-term to establish their individual risk for developing potential complications while optimizing their medical management. As the potential genetic etiology is explored further, it is important to screen first degree relatives for LV noncompaction or other cardiomyopathy forms. Studies have shown that 30% of screened family members are also diagnosed with LVNC or other types of cardiomyopathies [140]. In addition, identifying all affected and unaffected family members is necessary to define if the LVNC phenotype in affected (clinically and sub-clinically) patients is progressive, and whether proactive genetic counseling and individualized prevention and medical management should be initiated [19].

## 7. Genetic Testing

Genetic testing is not routinely performed in the clinical setting in many countries. As previously noted, patients with suspected LVNC based on the above diagnostic testing and clinical presentation should be considered for genetic testing, given LVNC’s strong correlation with genetic etiologies. Family members of affected individuals should also be genetically screened. Recent studies have shown the importance of genetic testing in this patient population due to potential adjustment of clinical management and risk stratification for family members via cascade testing [141, 142]. A broad cardiomyopathy genetic panel may be considered at time of presentation to identify LVNC pathologic variants, including those overlapping with other cardiomyopathies [142]. One study demonstrated half of adult and pediatric LVNC probands and relatives had an identified genetic mutation via a targeted panel containing 17 genes, which included *MYH7*, *MYBPC3*, *ACTC1*, *TPM1*, *CSRP3*, *TAZ*, *LDB3*, cardiac troponins (*TNNC1*, *TNNT2*, *TNNI3*), cardiac-regulatory myosin light chains (*MYL2, MYL3*), theletonin (*TCAP*), calsequestrin (*CASQ2*), calreticulin (*CALR3*), phospholamban (*PLN*), and lamin A/C (*LMNA*) [80]. Other studies have demonstrated the utility of whole exome sequencing in LVNC probands and their family members [19, 20, 21, 22]. Genetic testing and familial screening for LVNC are essential for diagnosis, prognosis, and future genetic counseling among affected families [120]. Overall, without current gold standard diagnostic and genetic testing criteria, the accurate assessment of genotype-phenotype associations in inherited LVNC cases in both pediatric and adult populations is difficult. This is further complicated by the heterogeneity among LVNC phenotypes and potential progression of LV dysfunction, particularly in pediatric patients over time [140]. Uniform diagnostic criteria in assessment of symptoms, cardiac imaging, and electrocardiogram applied to asymptomatic and symptomatic pediatric and adult populations may lead to a more accurate depiction of LVNC’s genetic architecture.

## 8. Prognosis and Treatment 

Prognosis among adult and pediatric LVNC patients is affected by individual phenotypes and the presence of an identified genetic mutation or ventricular dysfunction [120, 143, 144]. LVNC patients have a significant risk for complications, such as ventricular arrhythmias, systolic dysfunction with heart failure, cardioembolic events, and sudden cardiac death, which may occur in up to two-thirds of LVNC patients [3, 145, 146]. The worst outcomes are also associated with mitochondrial disorders, hereditary neuromuscular disorders, or chromosomal defects. These are more commonly found in pediatric LVNC patients, specifically infants [5, 9]. A meta-analysis demonstrated an overall mortality rate of 14% among adults with isolated LVNC [120]. There is an increased risk for heart failure, heart transplantation and death among LVNC subtypes with RCM, ACM, DCM, HCM, and undulating phenotypes in children and adults [6, 14, 139, 147]. Children with LVNC are more likely to have an identified genetic mutation and associated CHD [138]. Prior studies have also demonstrated increased risk for death or heart transplantation rates among LVNC patients with overlapping phenotypes compared to those with the isolated LVNC phenotype [6, 147]. Between 60% to 75% of LVNC patients either die or undergo cardiac transplantation within 6 years of diagnosis [6, 148, 149]; heart transplantation is more common in pediatric LVNC patients with a higher incidence of extracorporeal membrane oxygenation (ECMO) and inotropic use employed as a bridge to transplant, compared to those with idiopathic cardiomyopathy. Moreover, pediatric LVNC patients with associated CHD have worse postoperative outcomes following cardiac surgery and longer hospitalizations, compared to those with isolated CHD [150].

There is no specific therapy for LVNC except for consensus guideline-directed heart failure treatments for various cardiomyopathies and arrhythmias across age groups. Heart failure guideline-directed medical therapy (GDMT), including beta blocker, angiotensin-converting-enzyme (ACE) inhibitors, angiotensin II receptor blocker (ARB) and angiotensin receptor/neprilysin inhibitor (ARNI), has been shown to improve systolic function and favorable ventricular remodeling in adult LVNC patients [134]. Mineralocorticoid receptor antagonists (MRA) and sodium-glucose cotransporter 2 inhibitors (SGLT2i) are additional components of adult heart failure GDMT. Due to the limited pediatric data from single center studies, these medications are often prescribed based on pediatric heart failure expert guidance and extrapolation from the adult clinical trials [151, 152]. Cardiac resynchronization therapy is typically utilized only in the adult population [120]. Reduced systolic function and deep intertrabecular recesses may contribute to the increased risk of thrombosis formation. Chronic anticoagulation is generally recommended as primary prevention for thromboembolic events, such as strokes [138, 148]. The clinical necessity of therapeutic anticoagulation in benign cases of adult LV hypertrabeculation and pregnant women [153, 154]. If LVNC patients do not respond well to medical management, they should be evaluated for ventricular assist device placement or heart transplant as needed. Also, patients with significantly reduced ejection fraction or life-threatening arrhythmias should be considered for placement of an implantable cardioverter defibrillator (ICD) to prevent cardiac arrest and sudden cardiac death [94, 155].

Several critical differences in the management of pediatric LVNC patients with heart failure should be considered compared to adult patients. The majority of LVNC patients undergoing heart transplantation was pediatric, and their post-transplant survival was comparable with that of other cardiomyopathy patients [149]. Babies have the highest risk, and other risk factors for death or transplantation include female sex and severity of systolic dysfunction [147]. Although huge achievements have been made in diagnosis and treatment, limited quantifiable criteria may hinder early detection of LVNC and primary prevention of potential complications in newborns and young children [12, 133]. In addition, due to undeveloped capillary networks within the hyper-trabeculated meshwork and noncompacted endocardial islands, LVNC easily can be a substrate for ischemia and infarctions and thromboembolic events commonly displaying as peripheral embolism or stroke in pediatric LVNC cases [9]. Further secondary pathogenic processes, such as dissection of the myocardium, myocardial hypertrophy, or myocardial tearing caused by dilatation and hypervascularization, cause major adverse cardiac events and advanced deterioration of heart function [14]. Therefore, careful cardiorespiratory management with monitoring oxygen partial pressure, ventilation support, and medication therapy with beta blocker, ARB or ACE inhibitors are considered in pediatric LVNC patients with LV ejection fraction less than 45% [156, 157].

## 9. Conclusions 

Left ventricular noncompaction in children is a complex disease with heterogeneous phenotypes and a diverse array of associated genetic mutations. Children are more likely to have certain LVNC phenotypes, an identified genetic mutation, and heart transplantation compared to their adult counterparts. There are no widely accepted diagnostic criteria, but multiple image modalities are utilized to assist with the diagnosis and guide management. There is a wide spectrum of clinical presentations and long-term prognosis; therefore, patients diagnosed with childhood LVNC should be followed throughout their lifespan to optimize their medical management and prevent future complications based on their individual risk. Future studies are needed to establish gold standard diagnostic criteria and corroborate targeted therapies for this complex disease, especially in neonates and young pediatric populations.
